# A Geometric Approach to Average Problems on Multinomial and Negative Multinomial Models

**DOI:** 10.3390/e22030306

**Published:** 2020-03-08

**Authors:** Mingming Li, Huafei Sun, Didong Li

**Affiliations:** 1 School of Mathematics and Statistics, Beijing Institute of Technology, Beijing 100081, China; 3120160587@bit.edu.cn; 2 Beijing Key Laboratory on MCAACI, Beijing Institute of Technology, Beijing 100081, China; 3 Department of Mathematics, Duke University, Durham, NC 27708, USA; didongli@math.duke.edu

**Keywords:** structure characterization, average problem, geometric midpoints, Karcher mean

## Abstract

This paper is concerned with the formulation and computation of average problems on the multinomial and negative multinomial models. It can be deduced that the multinomial and negative multinomial models admit complementary geometric structures. Firstly, we investigate these geometric structures by providing various useful pre-derived expressions of some fundamental geometric quantities, such as Fisher-Riemannian metrics, α-connections and α-curvatures. Then, we proceed to consider some average methods based on these geometric structures. Specifically, we study the formulation and computation of the midpoint of two points and the Karcher mean of multiple points. In conclusion, we find some parallel results for the average problems on these two complementary models.

## 1. Introduction

The concept of an average of a set of points within a given geometric structure abounds in various mathematical research. Significant development has been produced since the conception of the Karcher mean [1]. Among the various structure to be studied, the standard simplex presents itself as an interesting framework since it can be directly connected to the parameter spaces of various probability distributions, such as the two to-be-discussed models—the multinomial and negative multinomial models. There are already some recent works involving the statistical modeling of the probability simplex [2]. In the present work, we provide alternative modelings by considering the multinomial and negative multinomial models with classical methods of information geometry.

As a newly developed theory, information geometry has supplied us with various measures of the discrepancy between any two probability distributions. In addition to some standard distance functions, divergence functions are intended for measuring the asymmetric proximity of probability distributions on an appropriate statistical model. Some geometric quantities, such as Riemannian metric and pair of dual connections, can be readily induced from a divergence function by its higher order derivatives [3,4]. In this way, the geometric structures of various parametric statistical models can be studied specifically [5,6,7], and the investigation about some particular parametric models can also be found in [8,9]. Among these models, we find the multinomial model and negative multinomial model especially interesting, in the sense that they can be seen as a pair of complementary model spaces. The multinomial model is well known as a spherical space of positive constant curvature [10], while the negative multinomial model is found to be a hyperbolic space of negative constant curvature [11].

To be more specific, the motivation of the present paper is twofold. Firstly, we aim at clarifying the complementary geometric structures of the multinomial and negative multinomial models. The main results about these geometric structures involving geometric quantities, such as Fisher-Riemannian metrics, α-connections and α-curvatures, are collected in Section 3, most of which can be derived in a standard way. In particular, this paper extends the isometric representation results about the multinomial model to those about the negative multinomial model, obtaining new insight into the complementary structures of these two models as illustrated by Table 1. Secondly, the original purpose of the formulation and computation of average problems is approached by utilizing these geometric structures. To this end, we propose a generalized concept of midpoints for two points and a computation scheme of Karcher mean for multiple points. For the midpoints, we generalize the Chernoff points in the literature [12] to some wider parametrized classes. For the Karcher mean, as there are many algorithmic results [13,14] for general manifolds, this paper mainly contributes to addressing some practical issues, such as initial point choice and iteration computation, which yields effective solving methods via the geometric structures within the multinomial model and negative multinomial model. The results about these average methods are presented in Section 4.

## 2. Preliminaries

For the sake of clarity, we summarize some preliminary knowledge about information divergence functions in this section (more details can be found in [15]).

Given a particular parametric statistical model $M=\{p_{\theta }\;|\;\theta \in \Theta \},$ for our purpose, here we mainly consider invariant divergences that satisfy the property of information monotonicity, as mentioned in [15]. A typical kind of invariant divergence is given in the form of the well-defined *f*-divergence as

$$D_{f}\left(p_{\theta }\;∥\;p_{\xi }\right)=\int_{x\in X}p_{\theta }\left(x\right)f\left(\frac{p_{\xi }\left(x\right)}{p_{\theta }\left(x\right)}\right)dx,$$

where *f* is a convex function satisfying f(1)=0 and $f^{\prime \prime }\left(1\right)=1$.

A commonly used class of *f*-divergences is given by the α-divergence $D^{\left(\alpha \right)}$, with

$$f\left(u\right)=\left\{\begin{array}{cc} \frac{4}{1-\alpha^{2}}\left(1-u^{\frac{1+\alpha }{2}}\right), & \alpha \neq \pm 1,\\ -\log u, & \alpha =-1,\\ u\log u, & \alpha =1. \end{array}\right.$$

Particularly, for α=−1, the divergence $D^{\left(-1\right)}$ is usually called Kullback–Leibler divergence, which we denote by $D_{KL}$; for α=0, $D^{\left(0\right)}$ is usually called squared Hellinger distance, which we denote by $H^{2}$; and for α=3, $D^{\left(3\right)}$ is often related to the chi-square statistic.

While our later results are mainly related to the α-divergence, for comparison, we briefly mention another *f*-divergence called exponential divergence (see §12.3.6 [6]), which we denote by E with $f\left(u\right)=\frac{1}{2}\log^{2}u$.

For any divergence function *D* on a statistical manifold, we can construct another divergence function $D^{*}$ called dual divergence by swapping the arguments as ([16])

$$D^{*}\left(p_{\theta }\;∥\;p_{\xi }\right)=D\left(p_{\xi }\;∥\;p_{\theta }\right).$$

The dual divergence of an *f*-divergence $D_{f}$ is again an *f*-divergence $D_{f}^{*}=D_{f^{*}},$ with $f^{*}\left(u\right)=uf\left(\frac{1}{u}\right)$. Particularly, the dual divergence of the α-divergence $D^{\left(\alpha \right)}$ just is $D^{\left(-\alpha \right)}$.

To be mentioned, as a divergence is generally not symmetric, a symmetric divergence $D_{s}$ can be constructed from an asymmetric *D* by averaging it with its dual:

$$D_{s}=\frac{1}{2}\left(D+D^{*}\right).$$

A Riemannian metric *g* can be induced by a divergence *D* as

(1) $$g\left(\frac{\partial }{\partial \theta_{i}},\frac{\partial }{\partial \theta_{j}}\right)=g_{ij}\left(\theta \right)={\left.\frac{\partial^{2}}{\partial \xi_{i}\partial \xi_{j}}\right|}_{\xi =\theta }D\left(p_{\theta }\;∥\;p_{\xi }\right),$$

which is equivalent to the usual Fisher-Riemannian metric in the case when *D* is an *f*-divergence.

Furthermore, an affine connection ∇ is induced by the divergence *D* with connection coefficients

(2) $$g\left(\nabla_{\frac{\partial }{\partial \theta_{i}}}\frac{\partial }{\partial \theta_{j}},\frac{\partial }{\partial \theta_{k}}\right)=\Gamma_{ijk}\left(\theta \right)=-{\left.\frac{\partial^{3}}{\partial \theta_{i}\partial \theta_{j}\partial \xi_{k}}\right|}_{\xi =\theta }D\left(p_{\theta }\;∥\;p_{\xi }\right).$$

Similarly, another affine connection $\nabla^{*}$ can be obtained by replacing *D* with the dual divergence $D^{*}$ in the above formula. Thus, with the primal connection ∇ and dual connection $\nabla^{*}$, the statistical manifold admits a dual structure $\left(M,g,\nabla ,\nabla^{*}\right)$. Furthermore, the structure $\left(M,g,\nabla ,\nabla^{*}\right)$ is called dually flat if both ∇ and $\nabla^{*}$ are flat.

As a well known result [15], the primal connection induced by an *f*-divergence $D_{f}$ is the same as the usual α-connection $\nabla^{\left(\alpha \right)}$ with $\alpha =3+2\;f^{\prime \prime \prime }\left(1\right),$ while the induced dual connection is $\nabla^{\left(-\alpha \right)}$. Particularly, one can check that the primal connection induced by the α-divergence $D^{\left(\alpha \right)}$ is exactly the α-connection $\nabla^{\left(\alpha \right)}$.

## 3. Geometric Structure of Multinomial and Negative Multinomial Models

### 3.1. Basic Information Geometric Structure

In this subsection, we present the parametric formulation of the multinomial and negative multinomial model, respectively. Then some basic results about divergences and geometric structures are derived for both models.

#### 3.1.1. Multinomial Model

Consider the multinomial *n*-dimensional model $M_{N}$ consisting of (*n* + 1)-nominal distributions with probability mass function given by

(3) $$p_{\theta }\left(x_{0},x_{1},\ldots ,x_{n}\right)=\frac{N!}{x_{0}!x_{1}!\cdots x_{n}!}\theta_{0}^{x_{0}}\theta_{1}^{x_{1}}\cdots \theta_{n}^{x_{n}},$$

where $\sum_{i=0}^{n}x_{i}=N$ and the parametrization is given by $\theta =(\theta_{1},\ldots ,\theta_{n})$ with $\theta_{0}=1-\sum_{i=1}^{n}\theta_{i}$.

We can rewrite Equation (3) as

$$p_{\theta }\left(x_{0},x_{1},\ldots ,x_{n}\right)=\frac{N!}{x_{0}!x_{1}!\cdots x_{n}!}\exp\left(\sum_{i=1}^{n}x_{i}\log\frac{\theta_{i}}{\theta_{0}}+N\log\theta_{0}\right).$$

By some general knowledge about exponential distribution families [17], we see that the multinomial model $M_{N}$ admits the natural parameters

(4) $$\eta_{i}=\log\frac{\theta_{i}}{\theta_{0}},\;i=1,\ldots ,n,$$

and the potential function

$$\psi \left(\theta \right)=-N\log\theta_{0}=N\log\left(1+\sum_{i=1}^{n}\exp\eta_{i}\right),$$

from which we also obtain the expectation parameters

(5) $$\partial \psi /\partial \eta_{i}=N{\left(1+\sum_{i=1}^{n}\exp\eta_{i}\right)}^{-1}\exp\eta_{i}=N\theta_{i},\;i=1,\ldots ,n.$$

Next, we have the following result by direct calculation.

**Proposition** **1.**  *The divergences introduced in Section 2 are obtained for $M_{N}$ as follows:*

*the Kullback–Leibler divergence*

$$D_{KL}\left(p_{\theta }\;∥\;p_{\xi }\right)=N\sum_{i=0}^{n}\theta_{i}\log\frac{\theta_{i}}{\xi_{i}},$$

*the squared Hellinger distance*

$$H^{2}\left(p_{\theta },p_{\xi }\right)=4\left[1-{\left(\sum_{i=0}^{n}\sqrt{\theta_{i}\;\xi_{i}}\right)}^{N}\right],$$

*the α-divergence*

$$D^{\left(\alpha \right)}\left(p_{\theta }\;∥\;p_{\xi }\right)=\frac{4}{1-\alpha^{2}}\left[1-{\left(\sum_{i=0}^{n}\theta_{i}^{\frac{1-\alpha }{2}}\xi_{i}^{\frac{1+\alpha }{2}}\right)}^{N}\right]$$

*and the exponential divergence*

$$E\left(p_{\theta }\;∥\;p_{\xi }\right)=\frac{N(N-1)}{2}{\left(\sum_{i=0}^{n}\theta_{i}\log\frac{\theta_{i}}{\xi_{i}}\right)}^{2}+\frac{N}{2}\sum_{i=0}^{n}\theta_{i}\log^{2}\frac{\theta_{i}}{\xi_{i}}.$$

From any one of these expressions, by using Equation (1), we obtain the Fisher-Riemannian metric matrix as

(6) $$g_{ij}\left(\theta \right)=\frac{N}{\theta_{0}}+\frac{N}{\theta_{i}}\delta_{ij},$$

where $\delta_{ij}=1$ if i=j and 0 otherwise. Then, the inverse matrix of *g* can also be obtained as

(7) $$g^{ij}\left(\theta \right)=\frac{\theta_{i}}{N}\delta_{ij}-\frac{\theta_{i}\theta_{j}}{N}.$$

Furthermore, by direct verification with the metric expression of Equation (6), we have the following well-known result.

**Theorem** **1**  ([18]). *An isometry is established between the multinomial model $M_{N}$ and the n-sphere within the non-negative orthant of the Euclidean space $R^{n+1}$ by the parametric mapping:*

(8) $$\left(\theta_{0},\theta_{1},\ldots ,\theta_{n}\right)\mapsto 2\sqrt{N}\left(\theta_{0}^{1/2},\theta_{1}^{1/2},\ldots ,\theta_{n}^{1/2}\right).$$

*Consequently, via this isometry, the Fisher-Riemannian geodesic distance between two parameters θ and ξ of $M_{N}$ is given by*

(9) $$d\left(\theta ,\xi \right)=2\sqrt{N}\arccos\left(\sum_{i=0}^{n}\sqrt{\theta_{i}\;\xi_{i}}\right).$$

Referring to some basic differential geometrical concepts (see [19]), we can derive some further consequent results as follows.

**Corollary** **1.**  *The multinomial n-dimensional model $M_{N}$ with the Fisher-Riemannian metric is a Riemannian manifold of constant sectional curvature $K=\frac{1}{4\;N}$ and scalar curvature $S=\frac{n(n-1)}{4\;N}$. Furthermore, for a unit speed geodesic γ in $M_{N}$, the normal Jacobi fields along γ are precisely linear combinations of the vector fields, which are in the form of $J\left(t\right)=\sin\left(2\sqrt{N}\;t\right)E\left(t\right)$ or $J\left(t\right)=\cos\left(2\sqrt{N}\;t\right)E\left(t\right)$, where E is any parallel normal vector field along γ.*

#### 3.1.2. Negative Multinomial Model

Consider the negative multinomial *n*-dimensional model ${\mathrm{NM}}_{M}$ consisting of negative (*n* + 1)-nominal distributions with probability mass function given by

(10) $$p_{\theta }\left(x_{1},\ldots ,x_{n}\right)=\frac{\Gamma (M+x_{1}+\cdots +x_{n})}{\Gamma \left(M\right)\;x_{1}!\cdots x_{n}!}\theta_{0}^{M}\theta_{1}^{x_{1}}\cdots \theta_{n}^{x_{n}},$$

where $M>0,\;x_{1},\ldots ,x_{n}\geq 0$ and the parametrization is given by $\theta =(\theta_{1},\ldots ,\theta_{n})$ with $\theta_{0}=1-\sum_{i=1}^{n}\theta_{i}$.

With the rewritten form of Equation (10) as

$$p_{\theta }\left(x_{1},\ldots ,x_{n}\right)=\frac{\Gamma (M+x_{1}+\cdots +x_{n})}{\Gamma \left(M\right)\;x_{1}!\cdots x_{n}!}\exp\left(\sum_{i=1}^{n}x_{i}\log\theta_{i}+M\log\theta_{0}\right),$$

we find that the negative multinomial model ${\mathrm{NM}}_{M}$ admits the natural parameters

(11) $$\eta_{i}=\log\theta_{i},\;i=1,\ldots ,n,$$

and the potential function

$$\psi \left(\theta \right)=-M\log\theta_{0}=-M\log\left(1-\sum_{i=1}^{n}\exp\eta_{i}\right),$$

from which we also obtain the expectation parameters

(12) $$\partial \psi /\partial \eta_{i}=M{\left(1-\sum_{i=1}^{n}\exp\eta_{i}\right)}^{-1}\exp\eta_{i}=M\;\frac{\theta_{i}}{\theta_{0}}.$$

Again, we derive the following result by direct calculation.

**Proposition** **2.**  *The divergences introduced in Section 2 are obtained for ${\mathrm{NM}}_{M}$ as follows:*

*the Kullback–Leibler divergence*

$$D_{KL}\left(p_{\theta }\;∥\;p_{\xi }\right)=M\log\frac{\theta_{0}}{\xi_{0}}+M\sum_{i=1}^{n}\frac{\theta_{i}}{\theta_{0}}\log\frac{\theta_{i}}{\xi_{i}},$$

*the squared Hellinger distance*

$$H^{2}\left(p_{\theta },p_{\xi }\right)=4\left[1-{\left(\frac{\sqrt{\theta_{0}\xi_{0}}}{1-\sum_{i=1}^{n}\sqrt{\theta_{i}\xi_{i}}}\right)}^{M}\right],$$

*the α-divergence*

$$D^{\left(\alpha \right)}\left(p_{\theta }\;∥\;p_{\xi }\right)=\frac{4}{1-\alpha^{2}}\left[1-{\left(\frac{\theta_{0}^{\frac{1-\alpha }{2}}\xi_{0}^{\frac{1+\alpha }{2}}}{1-\sum_{i=1}^{n}\theta_{i}^{\frac{1-\alpha }{2}}\xi_{i}^{\frac{1+\alpha }{2}}}\right)}^{M}\right]$$

*and the exponential divergence*

$$E\left(p_{\theta }\;∥\;p_{\xi }\right)=\frac{M^{2}}{2}{\left(\log\frac{\theta_{0}}{\xi_{0}}+\sum_{i=1}^{n}\frac{\theta_{i}}{\theta_{0}}\log\frac{\theta_{i}}{\xi_{i}}\right)}^{2}+\frac{M}{2}\left[\sum_{i=1}^{n}\frac{\theta_{i}}{\theta_{0}}\log^{2}\frac{\theta_{i}}{\xi_{i}}+{\left(\sum_{i=1}^{n}\frac{\theta_{i}}{\theta_{0}}\log\frac{\theta_{i}}{\xi_{i}}\right)}^{2}\right].$$

Next, applying Equation (1) to the divergences in the foregoing proposition, we obtain the Fisher-Riemannian metric matrix as

(13) $$g_{ij}=\frac{M}{\theta_{0}^{2}}+\frac{M}{\theta_{0}\theta_{i}}\delta_{ij},$$

and its inverse matrix as

(14) $$g^{ij}=\frac{\theta_{0}}{M}\left(\theta_{i}\delta_{ij}-\theta_{i}\theta_{j}\right).$$

Furthermore, by direct verification with the metric expression of Equation (13), we have the following result parallel to Theorem 1.

**Theorem** **2.**  *An isometry is established between the negative multinomial model ${\mathrm{NM}}_{M}$ and the n-hyperbola within the nonnegative orthant of the Minkowski space $R^{1,n}$ by the parametric mapping:*

(15) $$\left(\theta_{0},\theta_{1},\ldots ,\theta_{n}\right)\mapsto 2\sqrt{M}\theta_{0}^{-1/2}\left(1,\theta_{1}^{1/2},\ldots ,\theta_{n}^{1/2}\right).$$

*Consequently, via this isometry, the Fisher-Riemannian geodesic distance between two parameters θ and ξ of ${\mathrm{NM}}_{M}$ is given by*

(16) $$d\left(\theta ,\xi \right)=2\sqrt{M}\;\mathrm{arcosh}\left(\frac{1-\sum_{i=1}^{n}\sqrt{\theta_{i}\;\xi_{i}}}{\sqrt{\theta_{0}\xi_{0}}}\right).$$

Again, some further consequent results are obtained as follows.

**Corollary** **2.**  *The negative multinomial n-dimensional model ${\mathrm{NM}}_{M}$ with the Fisher-Riemannian metric is a Riemannian manifold of constant sectional curvature $K=-\frac{1}{4\;M}$ and scalar curvature $S=-\frac{n(n-1)}{4\;M}$. Furthermore, for a unit speed geodesic γ in ${\mathrm{NM}}_{M}$, the normal Jacobi fields along γ are precisely linear combinations of the vector fields, which are in the form of $J\left(t\right)=\sinh\left(2\sqrt{M}\;t\right)E\left(t\right)$ or $J\left(t\right)=\cosh\left(2\sqrt{M}\;t\right)E\left(t\right)$, where E is any parallel normal vector field along γ.*

### 3.2. Dual Structures

In this section, we derive the α-connection coefficients and curvatures of the multinomial and negative multinomial model. While some basic results are equivalent to those in [11], our calculation is performed directly with the original parameters in order to give a clear presentation of the results.

The α-connection coefficients can be obtained by applying the calculation of Equation (2) to the α-divergence $D^{\left(\alpha \right)}$, as mentioned in Section 2. However, an easier derivation of the α-connection $\nabla^{\left(\alpha \right)}$ is given in the form of a linear combination of the mixture connection $\nabla^{\left(m\right)}=\nabla^{\left(-1\right)}$ and the exponential connection $\nabla^{\left(e\right)}=\nabla^{\left(1\right)}$. With the mixture connection coefficients obtained from the Kullback–Leibler divergence as

(17) $$\Gamma_{ijk}^{\left(m\right)}\left(\theta \right)=-{\left.\frac{\partial^{3}}{\partial \theta_{i}\partial \theta_{j}\partial \xi_{k}}\right|}_{\xi =\theta }D_{KL}\left(p_{\theta }\;∥\;p_{\xi }\right),$$

and the exponential connection coefficients obtained from the dual Kullback–Leibler divergence as

(18) $$\Gamma_{ijk}^{\left(e\right)}\left(\theta \right)=-{\left.\frac{\partial^{3}}{\partial \theta_{i}\partial \theta_{j}\partial \xi_{k}}\right|}_{\xi =\theta }D_{KL}^{*}\left(p_{\theta }\;∥\;p_{\xi }\right),$$

the α-connection coefficients are given by

(19) $$\Gamma_{ijk}^{\left(\alpha \right)}\left(\theta \right)=\frac{1+\alpha }{2}\Gamma_{ijk}^{\left(e\right)}\left(\theta \right)+\frac{1-\alpha }{2}\Gamma_{ijk}^{\left(m\right)}\left(\theta \right).$$

Next, the α-curvature tensor $R^{\left(\alpha \right)}$ is defined by

$$R^{\left(\alpha \right)}\left(X,Y\right)Z=\nabla_{X}^{\left(\alpha \right)}\nabla_{Y}^{\left(\alpha \right)}Z-\nabla_{Y}^{\left(\alpha \right)}\nabla_{X}^{\left(\alpha \right)}Z-\nabla_{\left[X,Y\right]}^{\left(\alpha \right)}Z,$$

where [X,Y] denotes the Lie bracket of *X* and *Y*. By the duality condition (see [20])

$$Xg\left(Y,Z\right)=g\left(\nabla_{X}^{\left(\alpha \right)}Y,Z\right)+g\left(Y,\nabla_{X}^{\left(-\alpha \right)}Z\right),$$

one can check that the following identity holds:

(20) $$g\left(R^{\left(\alpha \right)}\left(\partial_{i},\partial_{j}\right)\partial_{j},\partial_{i}\right)=\partial_{i}\Gamma_{jji}^{\left(\alpha \right)}-\partial_{j}\Gamma_{iji}^{\left(\alpha \right)}+\sum_{k,l}g^{kl}\left(\Gamma_{ijk}^{\left(\alpha \right)}\Gamma_{jil}^{\left(-\alpha \right)}-\Gamma_{jjk}^{\left(\alpha \right)}\Gamma_{iil}^{\left(-\alpha \right)}\right),$$

where $\frac{\partial }{\partial \theta_{i}}$ is shortened as $\partial_{i}$.

At last, the α-sectional curvature spanned by two tangent vectors $\partial_{i}$ and $\partial_{j}$ (i≠j) is determined by

(21) $$K^{\left(\alpha \right)}\left(\partial_{i},\partial_{j}\right)=\frac{g\left(R^{\left(\alpha \right)}\left(\partial_{i},\partial_{j}\right)\partial_{j},\partial_{i}\right)}{g_{ii}g_{jj}-g_{ij}^{2}}.$$

#### 3.2.1. Multinomial Model

For the multinomial model $M_{N}$, by applying Equations (17)–(19) with the Kullback–Leibler divergence in Propositon 1, we have the mixture connection coefficients

$$\Gamma_{ijk}^{\left(m\right)}\left(\theta \right)=0,$$

the exponential connection coefficients

$$\Gamma_{ijk}^{\left(e\right)}\left(\theta \right)=\frac{N}{\theta_{0}^{2}}-\frac{N}{\theta_{i}^{2}}\delta_{ijk}$$

and the α-connection coefficients

$$\Gamma_{ijk}^{\left(\alpha \right)}\left(\theta \right)=\frac{1+\alpha }{2}\Gamma_{ijk}^{\left(e\right)}\left(\theta \right).$$

Furthermore, by Equations (6), (7) and (20), we obtain

$$g\left(R^{\left(\alpha \right)}\left(\partial_{i},\partial_{j}\right)\partial_{j},\partial_{i}\right)=\frac{(1-\alpha^{2})N}{4}\left(\frac{1}{\theta_{0}\theta_{i}}+\frac{1}{\theta_{0}\theta_{j}}+\frac{1}{\theta_{i}\theta_{j}}\right),$$

$$g_{ii}g_{jj}-g_{ij}^{2}=N^{2}\left(\frac{1}{\theta_{0}\theta_{i}}+\frac{1}{\theta_{0}\theta_{j}}+\frac{1}{\theta_{i}\theta_{j}}\right).$$

Thus, via Equation (21), we recover the following result.

**Theorem** **3**  ([11]). *The multinomial model $M_{N}$ admits constant α-sectional curvature*

(22) $$K^{\left(\alpha \right)}=\frac{1-\alpha^{2}}{4\;N}.$$

#### 3.2.2. Negative Multinomial Model

For the negative multinomial model ${\mathrm{NM}}_{M}$, by applying Equations(17)–(19) with the Kullback–Leibler divergence in Propositon 2, we have the mixture connection coefficients

$$\Gamma_{ijk}^{\left(m\right)}\left(\theta \right)=\frac{M}{\theta_{0}^{2}}\left(\frac{2}{\theta_{0}}+\frac{\delta_{ik}+\delta_{jk}}{\theta_{k}}\right)=\left(g_{ik}+g_{jk}\right)/\theta_{0},$$

the exponential connection coefficients

$$\Gamma_{ijk}^{\left(e\right)}\left(\theta \right)=-\frac{M}{\theta_{0}\theta_{i}^{2}}\delta_{ijk}-\frac{M}{\theta_{0}^{2}\theta_{i}}\delta_{ij}=-g_{ik}\delta_{ij}/\theta_{i}$$

and the α-connection coefficients

$$\Gamma_{ijk}^{\left(\alpha \right)}\left(\theta \right)=\frac{1+\alpha }{2}\Gamma_{ijk}^{\left(e\right)}\left(\theta \right)+\frac{1-\alpha }{2}\Gamma_{ijk}^{\left(m\right)}\left(\theta \right).$$

Furthermore, by Equations (13), (14) and (20), we have

$$g\left(R^{\left(\alpha \right)}\left(\partial_{i},\partial_{j}\right)\partial_{j},\partial_{i}\right)=-\frac{(1-\alpha^{2})M}{4\;\theta_{0}^{2}}\left(\frac{1}{\theta_{0}\theta_{i}}+\frac{1}{\theta_{0}\theta_{j}}+\frac{1}{\theta_{i}\theta_{j}}\right),$$

$$g_{ii}g_{jj}-g_{ij}^{2}=\frac{M^{2}}{\theta_{0}^{2}}\left(\frac{1}{\theta_{0}\theta_{i}}+\frac{1}{\theta_{0}\theta_{j}}+\frac{1}{\theta_{i}\theta_{j}}\right).$$

Again, via Equation (21), we recover another parallel result.

**Theorem** **4**  ([11]). *The negative multinomial model ${\mathrm{NM}}_{M}$ admits constant α-sectional curvature*

(23) $$K^{\left(\alpha \right)}=-\frac{1-\alpha^{2}}{4\;M}.$$

For clarity, we summarize these results about the complementary geometric structures of the multinomial and negative multinomial models in Table 1.

## 4. Geometric Average Methods on Multinomial and Negative Multinomial Models

In this section, we present some average methods induced by the geometry of the multinomial and negative multinomial models.

Firstly, we consider the particular case when the to-be-averaged set consists of only two points. In this case, the problem is to find a method of computing a midpoint in some geometric sense. Next, via some techniques related to the Karcher mean, we consider the general case with a set of multiple points.

### 4.1. Midpoints of Two Points

In this subsection, again within the multinomial and negative multinomial model, we study a particular class of midpoints named Chernoff points. The original Chernoff point, which is motivated by the application of computing the best error exponent for the Bayesian hypothesis testing problem, is determined as the intersection point of an exponential geodesic and a mixture bisector [21]. Furthermore, there are three other generalized Chernoff points proposed by [12].

To present a further generalization, here we formulate the concepts of α-geodesic and α-bisector determined by two probability distributions $p_{\theta^{\prime }}$ and $p_{\theta^{\prime \prime }}$ of a parametric statistical model M.

**Definition** **1.**  *The α-geodesic is determined by the geodesic equation of the α-connection $\nabla^{\left(\alpha \right)}$ as*

$$G^{\left(\alpha \right)}\left(p_{\theta^{\prime }},p_{\theta^{\prime \prime }}\right)=\left\{p_{\theta (t)}\in M\left|\;\theta \left(0\right)=\theta^{\prime },\;\theta \left(1\right)=\theta^{\prime \prime },\;\nabla_{\dot{\theta }\left(t\right)}^{\left(\alpha \right)}\dot{\theta }\left(t\right)=0,\;t\in \left[0,1\right]\right.\right\},$$

*where $\dot{\theta }\left(t\right)$ denotes the velocity vector of a curve θ(t).*

Particularly, since an exponential family model is ±1-flat, as can be directly seen from Equations (22) and (23) for our case, the exponential geodesic for α=1 can be determined by the linear interpolation of the natural parameters, while the mixture geodesic for α=−1 can be determined by the linear interpolation of the expectation parameters.

**Definition** **2.**  *The α-bisector is determined by the equi-divergence identity of the α-divergence $D^{\left(\alpha \right)}$ as*

(24) $${\mathrm{Bi}}^{\left(\alpha \right)}\left(p_{\theta^{\prime }},p_{\theta^{\prime \prime }}\right)=\left\{p_{\theta }\in M\left|\;D^{\left(\alpha \right)}\left(p_{\theta }\;∥\;p_{\theta^{\prime }}\right)=D^{\left(\alpha \right)}\left(p_{\theta }\;∥\;p_{\theta^{\prime \prime }}\right)\right.\right\}.$$

Particularly, the exponential bisector for α=1 is determined in terms of the dual Kullback–Leibler divergence, while the mixture bisector for α=−1 is determined in terms of the Kullback–Leibler divergence.

Then, we can generalize the notion of Chernoff points suggested by [12] as follows.

**Definition** **3.**  *Two types of generalized Chernoff points are given by the intersection points with parameter α:*

$$CP_{I}^{\left(\alpha \right)}\left(p_{\theta^{\prime }},p_{\theta^{\prime \prime }}\right)=G^{\left(\alpha \right)}\left(p_{\theta^{\prime }},p_{\theta^{\prime \prime }}\right)\cap {\mathrm{Bi}}^{\left(-\alpha \right)}\left(p_{\theta^{\prime }},p_{\theta^{\prime \prime }}\right),$$

$$CP_{\mathrm{II}}^{\left(\alpha \right)}\left(p_{\theta^{\prime }},p_{\theta^{\prime \prime }}\right)=G^{\left(\alpha \right)}\left(p_{\theta^{\prime }},p_{\theta^{\prime \prime }}\right)\cap {\mathrm{Bi}}^{\left(\alpha \right)}\left(p_{\theta^{\prime }},p_{\theta^{\prime \prime }}\right).$$

Thus, the Chernoff points already proposed in previous works can be recovered by setting α=±1 in Definition 3.

The existence of these intersection points is assured by the intermediate value property of the determining Equation (24), since replacing θ by $\theta^{\prime }$ and $\theta^{\prime \prime }$, respectively, we get two opposite inequalities due to the non-negativity of the α-divergence.

While the uniqueness can be proven for an exponential family model if α=±1 (see [12]), we conjecture it still holds for general cases but this is not pursued in the present paper.

To elucidate what we have mentioned earlier about the application of the original Chernoff point ($CP_{I}^{\left(1\right)}$ in our notation) to the binary Bayesian hypothesis testing problem, we present here the upper bound of the probability of error of the Bayesian decision suggested by [21] as

(25) $$\exp\left(-D_{KL}\left(p_{\theta^{*}}\;∥\;p_{\theta^{\prime }}\right)\right)=\exp\left(-D_{KL}\left(p_{\theta^{*}}\;∥\;p_{\theta^{\prime \prime }}\right)\right),$$

where $p_{\theta^{*}}$ denotes the Chernoff point $CP_{I}^{\left(1\right)}\left(p_{\theta^{\prime }},p_{\theta^{\prime \prime }}\right)$.

The overlapping case of the two classes of generalized Chernoff points is given by α=0. For the multinomial and negative multinomial model, by comparing the 0-divergence, i.e., squared Hellinger distance, with the Fisher-Riemannian geodesic distance presented in Section 3.1, we find that the generalized Chernoff point $CP_{I}^{\left(0\right)}\left(p_{\theta^{\prime }},p_{\theta^{\prime \prime }}\right)=CP_{\mathrm{II}}^{\left(0\right)}\left(p_{\theta^{\prime }},p_{\theta^{\prime \prime }}\right)$ is exactly the unique Fisher-Riemannian geodesic midpoint between $p_{\theta^{\prime }}$ and $p_{\theta^{\prime \prime }}$ within both models.

Next, we summarize some specific results about Chernoff points for both models as follows.

#### 4.1.1. Multinomial Model

For the multinomial model $M_{N}$, although the geodesic equation of the α-geodesic can be explicitly given in general cases, there are simple closed-form geodesic expressions at least for α=±1.

**Proposition** **3.**  *The exponential and mixture geodesics connecting two probability distributions $p_{\theta^{\prime }}$ and $p_{\theta^{\prime \prime }}$ of the multinomial model $M_{N}$ are given by*

$$G^{\left(1\right)}\left(p_{\theta^{\prime }},p_{\theta^{\prime \prime }}\right)=\left\{p_{\theta (t)}\in M_{N}\left|\;\theta_{i}\left(t\right)=\frac{{\left(\theta_{i}^{\prime }\right)}^{1-t}{\left(\theta_{i}^{\prime \prime }\right)}^{t}}{\sum_{i=0}^{n}{\left(\theta_{i}^{\prime }\right)}^{1-t}{\left(\theta_{i}^{\prime \prime }\right)}^{t}},\;i=0,\ldots ,n,\;t\in \left[0,1\right]\right.\right\},$$

$$G^{\left(-1\right)}\left(p_{\theta^{\prime }},p_{\theta^{\prime \prime }}\right)=\left\{p_{\theta (t)}\in M_{N}\left|\;\theta_{i}\left(t\right)=\left(1-t\right)\;\theta_{i}^{\prime }+t\;\theta_{i}^{\prime \prime },\;i=0,\ldots ,n,\;t\in \left[0,1\right]\right.\right\}.$$

**Proof.**  As already mentioned, the exponential and mixture geodesics can be easily obtained by the linear interpolation of the natural and expectation parameters by Equations (4) and (5), respectively. □

By using the expressions of α-divergences presented in Proposition 1, the α-bisectors are directly obtained as follows.

**Proposition** **4.**  *The α-bisectors between $p_{\theta^{\prime }}$ and $p_{\theta^{\prime \prime }}$ within the multinomial model $M_{N}$ are given by the following equations:*

$${\mathrm{Bi}}^{\left(\alpha \right)}\left(p_{\theta^{\prime }},p_{\theta^{\prime \prime }}\right)=\left\{p_{\theta }\in M_{N}\left|\;\sum_{i=0}^{n}\theta_{i}^{\frac{1-\alpha }{2}}[{\left(\theta_{i}^{\prime }\right)}^{\frac{1+\alpha }{2}}-{\left(\theta_{i}^{\prime \prime }\right)}^{\frac{1+\alpha }{2}}\right.]=0\right\},\;\alpha \neq \pm 1,$$

$${\mathrm{Bi}}^{\left(1\right)}\left(p_{\theta^{\prime }},p_{\theta^{\prime \prime }}\right)=\left\{p_{\theta }\in M_{N}\left|\;\sum_{i=0}^{n}\left(\theta_{i}^{\prime }-\theta_{i}^{\prime \prime }\right)\log\theta_{i}=\sum_{i=0}^{n}\left(\theta_{i}^{\prime }\log\theta_{i}^{\prime }-\theta_{i}^{\prime \prime }\log\theta_{i}^{\prime \prime }\right)\right.\right\},$$

$${\mathrm{Bi}}^{\left(-1\right)}\left(p_{\theta^{\prime }},p_{\theta^{\prime \prime }}\right)=\left\{p_{\theta }\in M_{N}\left|\;\sum_{i=0}^{n}\theta_{i}\log\frac{\theta_{i}^{\prime }}{\theta_{i}^{\prime \prime }}=0\right.\right\}.$$

Combining the previous two propositions, we have the following result about the determining equations for the four particular Chernoff points with α=±1.

**Theorem** **5.**  *The determining equations for the Chernoff points with α=±1 of $p_{\theta^{\prime }}$ and $p_{\theta^{\prime \prime }}$ within the multinomial model $M_{N}$ are expressed in terms of the argument t∈[0,1] of the corresponding geodesics as follows:*

*1.*  *For $CP_{I}^{\left(1\right)}\left(p_{\theta^{\prime }},p_{\theta^{\prime \prime }}\right)$,*
  $$\sum_{i=0}^{n}{\left(\theta_{i}^{\prime }\right)}^{1-t}{\left(\theta_{i}^{\prime \prime }\right)}^{t}\log\frac{\theta_{i}^{\prime }}{\theta_{i}^{\prime \prime }}=0;$$
*2.*  *For $CP_{I}^{\left(-1\right)}\left(p_{\theta^{\prime }},p_{\theta^{\prime \prime }}\right)$,*
  $$\sum_{i=0}^{n}\left(\theta_{i}^{\prime }-\theta_{i}^{\prime \prime }\right)\log\left[\left(1-t\right)\;\theta_{i}^{\prime }+t\;\theta_{i}^{\prime \prime }\right]=\sum_{i=0}^{n}\left(\theta_{i}^{\prime }\log\theta_{i}^{\prime }-\theta_{i}^{\prime \prime }\log\theta_{i}^{\prime \prime }\right);$$
*3.*  *For $CP_{\mathrm{II}}^{\left(1\right)}\left(p_{\theta^{\prime }},p_{\theta^{\prime \prime }}\right)$,*
  $$t=\frac{D_{KL}\left(p_{\theta^{\prime \prime }}\;∥\;p_{\theta^{\prime }}\right)}{D_{KL}\left(p_{\theta^{\prime }}\;∥\;p_{\theta^{\prime \prime }}\right)+D_{KL}\left(p_{\theta^{\prime \prime }}\;∥\;p_{\theta^{\prime }}\right)};$$
*4.*  *For $CP_{\mathrm{II}}^{\left(-1\right)}\left(p_{\theta^{\prime }},p_{\theta^{\prime \prime }}\right)$,*
  $$t=\frac{D_{KL}\left(p_{\theta^{\prime }}\;∥\;p_{\theta^{\prime \prime }}\right)}{D_{KL}\left(p_{\theta^{\prime }}\;∥\;p_{\theta^{\prime \prime }}\right)+D_{KL}\left(p_{\theta^{\prime \prime }}\;∥\;p_{\theta^{\prime }}\right)}.$$

With the Kullback–Leibler divergence $D_{KL}$ given by Propositon 1, the two points of second type $CP_{\mathrm{II}}^{\left(1\right)}$ and $CP_{\mathrm{II}}^{\left(-1\right)}$ are already in an explicit form. Whereas, the two points of first type $CP_{I}^{\left(1\right)}$ and $CP_{I}^{\left(-1\right)}$ are to be solved by using some numerical methods such as simple bisection as suggested in [12].

For the Fisher-Riemannian geodesic midpoint, we have the following result.

**Theorem** **6.**  The Fisher-Riemannian geodesic midpoint $p_{\theta^{*}}$ between $p_{\theta^{\prime }}$ and $p_{\theta^{\prime \prime }}$ in the multinomial model $M_{N}$ is determined by

$$\theta_{i}^{*}=\frac{{\left(\sqrt{\theta_{i}^{\prime }}+\sqrt{\theta_{i}^{\prime \prime }}\;\right)}^{2}}{2+2\sum_{i=0}^{n}\sqrt{\theta_{i}^{\prime }\theta_{i}^{\prime \prime }}},\;i=0,\ldots ,n.$$

**Proof.**  Let *F* be the isometry given by Equation (8). Denote the linear midpoint of the two image points $\frac{F\left(\theta^{\prime }\right)+F\left(\theta^{\prime \prime }\right)}{2}$ by $x^{*}\in R^{n+1}$. Then we normalize $x^{*}$ to the *n*-sphere as $u^{*}=2\sqrt{N}\;x^{*}/{∥\;x^{*}∥}_{e}$, where the Euclidean norm ${∥x∥}_{e}={\left(\sum_{i=0}^{n}x_{i}^{2}\right)}^{1/2}$ is used. At last, the required midpoint is obtained as the inverse image point $\theta^{*}=F^{-1}\left(u^{*}\right)$. □

To illustrate these notions for the multinomial model $M_{N}$, we present a numerical example as follows. The two parameters $\theta^{\prime }$ and $\theta^{\prime \prime }$ are taken as the empirical probability vectors of the first and second 100 decimal digits of π, respectively. The parameters of these two points and the resulting Chernoff points are summarized in Table 2.

As we can see, the pair of points $CP_{I}^{\left(1\right)}$ and $CP_{\mathrm{II}}^{\left(1\right)}$ admits a certain similarity between them as both of them lie on the same exponential geodesic, and so does the pair of points $CP_{I}^{\left(-1\right)}$ and $CP_{\mathrm{II}}^{\left(-1\right)}$ as both of them lie on the same mixture geodesic. Whereas, the Fisher-Riemannian geodesic midpoint $CP^{\left(0\right)}$ can be considered as a medium version among these Chernoff points.

To be mentioned particularly, the upper bound of the probability of error given by Equation (25) is obtained via $CP_{I}^{\left(1\right)}$ as being equal to $0.9862^{N}$. Thus, we can choose sufficiently large *N* so that the probability of error is less than some threshold value.

#### 4.1.2. Negative Multinomial Model

For the negative multinomial model ${\mathrm{NM}}_{M}$, again we can give the geodesic expressions for the exponential and mixture cases.

**Proposition** **5.**  *The exponential and mixture geodesics connecting two probability distributions $p_{\theta^{\prime }}$ and $p_{\theta^{\prime \prime }}$ of the negative multinomial model ${\mathrm{NM}}_{M}$ are given by*

$$G^{\left(1\right)}\left(p_{\theta^{\prime }},p_{\theta^{\prime \prime }}\right)=\left\{p_{\theta (t)}\in {\mathrm{NM}}_{M}\left|\;\theta_{i}\left(t\right)={\left(\theta_{i}^{\prime }\right)}^{1-t}{\left(\theta_{i}^{\prime \prime }\right)}^{t},\;i=1,\ldots ,n,\;t\in \left[0,1\right]\right.\right\},$$

$$G^{\left(-1\right)}\left(p_{\theta^{\prime }},p_{\theta^{\prime \prime }}\right)=\left\{p_{\theta (t)}\in {\mathrm{NM}}_{M}\left|\;\theta_{i}\left(t\right)=\frac{\left(1-t\right)\frac{\theta_{i}^{\prime }}{\theta_{0}^{\prime }}+t\frac{\theta_{i}^{\prime \prime }}{\theta_{0}^{\prime \prime }}}{\frac{1-t}{\theta_{0}^{\prime }}+\frac{t}{\theta_{0}^{\prime \prime }}},\;i=1,\ldots ,n,\;t\in \left[0,1\right]\right.\right\}.$$

**Proof.**  By using Equations (11) and (12), the exponential and mixture geodesics can be easily obtained by the linear interpolation of the natural and expectation parameters, respectively. □

By using the expressions of α-divergences presented in Proposition 2, the α-bisectors are directly obtained as follows.

**Proposition** **6.**  *The α-bisectors between $p_{\theta^{\prime }}$ and $p_{\theta^{\prime \prime }}$ within the negative multinomial model ${\mathrm{NM}}_{M}$ are given by the following equations:*

$${\mathrm{Bi}}^{\left(\alpha \right)}\left(p_{\theta^{\prime }},p_{\theta^{\prime \prime }}\right)=\left\{p_{\theta }\in M_{N}\left|\;\sum_{i=1}^{n}\theta_{i}^{\frac{1-\alpha }{2}}\left[{\left(\frac{\theta_{i}^{\prime }}{\theta_{0}^{\prime }}\right)}^{\frac{1+\alpha }{2}}-{\left(\frac{\theta_{i}^{\prime \prime }}{\theta_{0}^{\prime \prime }}\right)}^{\frac{1+\alpha }{2}}\right]={\left(\theta_{0}^{\prime }\right)}^{-\frac{1+\alpha }{2}}-{\left(\theta_{0}^{\prime \prime }\right)}^{-\frac{1+\alpha }{2}}\right.\right\},$$

$${\mathrm{Bi}}^{\left(1\right)}\left(p_{\theta^{\prime }},p_{\theta^{\prime \prime }}\right)=\left\{p_{\theta }\in {\mathrm{NM}}_{M}\left|\;\sum_{i=1}^{n}\left(\frac{\theta_{i}^{\prime }}{\theta_{0}^{\prime }}-\frac{\theta_{i}^{\prime \prime }}{\theta_{0}^{\prime \prime }}\right)\log\theta_{i}=\sum_{i=0}^{n}\left(\frac{\theta_{i}^{\prime }}{\theta_{0}^{\prime }}\log\theta_{i}^{\prime }-\frac{\theta_{i}^{\prime \prime }}{\theta_{0}^{\prime \prime }}\log\theta_{i}^{\prime \prime }\right)\right.\right\},$$

$${\mathrm{Bi}}^{\left(-1\right)}\left(p_{\theta^{\prime }},p_{\theta^{\prime \prime }}\right)=\left\{p_{\theta }\in {\mathrm{NM}}_{M}\left|\;\sum_{i=0}^{n}\theta_{i}\log\frac{\theta_{i}^{\prime }}{\theta_{i}^{\prime \prime }}=0\right.\right\}.$$

Combining the previous two propositions, we have the following result about the determining equations for the four particular Chernoff points with α=±1.

**Theorem** **7.**  *The determining equations for the Chernoff points with α=±1 of $p_{\theta^{\prime }}$ and $p_{\theta^{\prime \prime }}$ within the negative multinomial model ${\mathrm{NM}}_{M}$ are expressed in terms of the argument t∈[0,1] of the corresponding geodesics as follows:*

*1.*  *For $CP_{I}^{\left(1\right)}\left(p_{\theta^{\prime }},p_{\theta^{\prime \prime }}\right)$,*
  $$\log\frac{\theta_{0}^{\prime }}{\theta_{0}^{\prime \prime }}+\sum_{i=1}^{n}{\left(\theta_{i}^{\prime }\right)}^{1-t}{\left(\theta_{i}^{\prime \prime }\right)}^{t}\left(\log\frac{\theta_{i}^{\prime }}{\theta_{i}^{\prime \prime }}-\log\frac{\theta_{0}^{\prime }}{\theta_{0}^{\prime \prime }}\right)=0;$$
*2.*  *For $CP_{I}^{\left(-1\right)}\left(p_{\theta^{\prime }},p_{\theta^{\prime \prime }}\right)$,*
  $$\sum_{i=1}^{n}\left(\frac{\theta_{i}^{\prime }}{\theta_{0}^{\prime }}-\frac{\theta_{i}^{\prime \prime }}{\theta_{0}^{\prime \prime }}\right)\log\frac{\left(1-t\right)\frac{\theta_{i}^{\prime }}{\theta_{0}^{\prime }}+t\frac{\theta_{i}^{\prime \prime }}{\theta_{0}^{\prime \prime }}}{\frac{1-t}{\theta_{0}^{\prime }}+\frac{t}{\theta_{0}^{\prime \prime }}}=\sum_{i=0}^{n}\left(\frac{\theta_{i}^{\prime }}{\theta_{0}^{\prime }}\log\theta_{i}^{\prime }-\frac{\theta_{i}^{\prime \prime }}{\theta_{0}^{\prime \prime }}\log\theta_{i}^{\prime \prime }\right);$$
*3.*  *For $CP_{\mathrm{II}}^{\left(1\right)}\left(p_{\theta^{\prime }},p_{\theta^{\prime \prime }}\right)$,*
  $$t=\frac{D_{KL}\left(p_{\theta^{\prime \prime }}\;∥\;p_{\theta^{\prime }}\right)}{D_{KL}\left(p_{\theta^{\prime }}\;∥\;p_{\theta^{\prime \prime }}\right)+D_{KL}\left(p_{\theta^{\prime \prime }}\;∥\;p_{\theta^{\prime }}\right)};$$
*4.*  *For $CP_{\mathrm{II}}^{\left(-1\right)}\left(p_{\theta^{\prime }},p_{\theta^{\prime \prime }}\right)$,*
  $$t=\frac{D_{KL}\left(p_{\theta^{\prime }}\;∥\;p_{\theta^{\prime \prime }}\right)}{D_{KL}\left(p_{\theta^{\prime }}\;∥\;p_{\theta^{\prime \prime }}\right)+D_{KL}\left(p_{\theta^{\prime \prime }}\;∥\;p_{\theta^{\prime }}\right)}.$$

As can be seen, the two points of second type $CP_{\mathrm{II}}^{\left(1\right)}$ and $CP_{\mathrm{II}}^{\left(-1\right)}$ are in the same explicit form as before, except that the Kullback–Leibler divergence $D_{KL}$ is given by Proposition 2. And again, the two points of first type $CP_{I}^{\left(1\right)}$ and $CP_{I}^{\left(-1\right)}$ can be solved by using numerical methods.

For the Fisher-Riemannian geodesic midpoint, we have the following result being complementary to the previous one.

**Theorem** **8.**  The Fisher-Riemannian geodesic midpoint $p_{\theta^{*}}$ between $p_{\theta^{\prime }}$ and $p_{\theta^{\prime \prime }}$ in the negative multinomial model ${\mathrm{NM}}_{M}$ is determined by

$$\theta_{i}^{*}={\left[\frac{{\left(\theta_{0}^{\prime }\right)}^{-1/2}{\left(\theta_{i}^{\prime }\right)}^{1/2}+{\left(\theta_{0}^{\prime \prime }\right)}^{-1/2}{\left(\theta_{i}^{\prime \prime }\right)}^{1/2}}{{\left(\theta_{0}^{\prime }\right)}^{-1/2}+{\left(\theta_{0}^{\prime \prime }\right)}^{-1/2}}\right]}^{2},\;i=1,\ldots ,n.$$

**Proof.**  Let *F* be the isometry given by Equation (15). Denote the linear midpoint of the two image points $\frac{F\left(\theta^{\prime }\right)+F\left(\theta^{\prime \prime }\right)}{2}$ by $x^{*}\in R^{1,n}$. Then we normalize $x^{*}$ to the *n*-hyperbola as $u^{*}=2\sqrt{M}\;x^{*}/{∥\;x^{*}∥}_{m}$, where the Minkowski norm ${∥x∥}_{m}={\left(x_{0}^{2}-\sum_{i=1}^{n}x_{i}^{2}\right)}^{1/2}$ is used. At last, the required midpoint is obtained as the inverse image point $\theta^{*}=F^{-1}\left(u^{*}\right)$. □

A numerical illustration for the negative multinomial model ${\mathrm{NM}}_{M}$ is presented as follows. The two parameters $\theta^{\prime }$ and $\theta^{\prime \prime }$ are taken as the empirical probability vectors of the decimal digits of π within the first and second 10 appearances of “0”, respectively. The parameters of these two points and the resulting Chernoff points are summarized in Table 3.

Again, the pair of points $CP_{I}^{\left(1\right)}$ and $CP_{\mathrm{II}}^{\left(1\right)}$ admits a certain similarity between them, and so does the pair of points $CP_{I}^{\left(-1\right)}$ and $CP_{\mathrm{II}}^{\left(-1\right)}$. Whereas, the Fisher-Riemannian geodesic midpoint $CP^{\left(0\right)}$ serves as a medium version among these Chernoff points.

The upper bound of the probability of error given by Equation (25) is obtained via $CP_{I}^{\left(1\right)}$ as being equal to $0.8789^{M}$. Thus, sufficiently large *M* need to be chosen so that the probability of error is less than some threshold value.

### 4.2. Karcher Means of Multiple Points

A natural generalization of the Fisher-Riemannian geodesic midpoint between two points is given by the Karcher mean among multiple points.

Let M be a metric space and *S* be a set of points on M. Define a criterion function f:M→R by

$$f\left(x\right):=\frac{1}{2\;|S|}\sum_{p\in S}d{\left(x,p\right)}^{2},$$

where d(·,·) is the distance function and |S| is the number of points of *S*. If the minimizer of the function *f* exists and is unique, then it is called the *Karcher mean* of *S* on M. If *d* is a Riemannian metric on M, then the negative gradient vector field with respect to *f* is found to be the usual average of the corresponding points in the tangent space ([1]):

(26) $$-\nabla f\left(x\right)=\frac{1}{\left|S\right|}\sum_{p\in S}\exp_{x}^{-1}\left(p\right),$$

where $\exp_{x}^{-1}$ is the inverse of the Riemannian exponential map at *x*. In view of this, the Karcher mean can be alternatively understood as a point at which the above vector field vanishes.

The Karcher mean may be not unique unless all points are located on a geodesically convex region. For example, there are infinitely many geodesic midpoints between two antipodal points on a sphere. However, for model spaces, such as open half-sphere and hyperbolic space, there are existing results to assure the existence and uniqueness of Karcher mean ([22]). Thus, by virtue of Theorem 1 and Theorem 2, we conclude that the concept of a Karcher mean is well-defined on the multinomial and negative multinomial models.

Now, we focus ourselves on the computation of the Karcher mean on these two models. The Karcher mean of two points admits a closed-form expression as the Fisher-Riemannian geodesic midpoint presented previously, but for multiple points, we can only expect to obtain a numerical solution of the Karcher mean.

By virtue of Equation (26), there is a Riemannian gradient iteration algorithm with a locally superlinear convergence in general ([23]):

$$x_{i+1}=\exp_{x_{i}}\left(\frac{1}{\left|S\right|}\sum_{p\in S}\exp_{x_{i}}^{-1}\left(p\right)\right).$$

However, this general algorithm is difficult to apply in practice unless proper representations of the models are derived. In our case, as we have prepared enough geometric representation results within the multinomial and negative multinomial models in Section 3, we still have to address two practical issuses: the choice of initial points and the computation of Riemannian exponential map $\exp_{x_{i}}$ and its inverse $\exp_{x_{i}}^{-1}$.

#### 4.2.1. Initial Points

Let *S* be a set of parameters to be averaged in either the model $M_{N}$ or ${\mathrm{NM}}_{M}$, we present a heuristic approach motivated by the proof of Theorem 1 and Theorem 2 to provide an initial point choice. The main procedure is presented as follows (here N=M=1 is assumed as basic ideas are unchanged up to scale):

1. Set the average of isometry images $x^{*}:=\frac{1}{\left|S\right|}\sum_{\theta \in S}F\left(\theta \right)$;
2. Set the normalized vector $u^{*}:=2\;x^{*}/∥\;x^{*}∥$; the parameter of the initial point is given by $\theta^{*}:=F^{-1}\left(u^{*}\right)$.

For the model $M_{N}$, the isometry *F* is given by Equation (8), and the norm ∥·∥ is the Euclidean norm ${∥\cdot ∥}_{e}$ in the proof of Theorem 1. For the model ${\mathrm{NM}}_{M}$, the isometry *F* is given by Equation (15), and the norm ∥·∥ is the Minkowski norm ${∥\cdot ∥}_{m}$ in the proof of Theorem 2.

#### 4.2.2. Computation of Riemannian Exponential and its Inverse

Within each of the model $M_{N}$ and ${\mathrm{NM}}_{M}$ (again N=M=1 is assumed), the Riemannian exponential and its inverse map can be computed in an easy-to-manipulate way. Except for the isometry *F* and the norm ∥·∥ being given as before, we also need to set 〈·,·〉 by $\langle x,y\rangle =\sum_{i=0}^{n}x_{i}y_{i}$ as the Euclidean inner product for the model $M_{N}$ and by $\langle x,y\rangle =x_{0}y_{0}-\sum_{i=1}^{n}x_{i}y_{i}$ as the Minkowski inner product for the model ${\mathrm{NM}}_{M}$.

To compute $\exp_{\theta }^{-1}\left(\xi \right)$, we have the following steps:

1. Set a tangent vector by orthogonal projection
  x:=F(ξ)−F(θ)〈F(ξ),F(θ)〉/4;
2. Scale the vector by geodesic distance
  u:=d(θ,ξ)x/∥x∥,
  where the geodesic distance d(·,·) is given by Equation (9) for $M_{N}$ and by Equation (16) for ${\mathrm{NM}}_{M}$.

Thus, we use the resulting vector *u* to represent $\exp_{\theta }^{-1}\left(\xi \right)$.

To compute $\exp_{\theta }\left(u\right)$, we need to:

1. Set the angle α:=∥u∥/2;
2. For $M_{N}$, express the corresponding point on the sphere
  $$x:=F\left(\theta \right)\cos\alpha +{2\;∥u∥}^{-1}\;u\sin\alpha ,$$
  for ${\mathrm{NM}}_{M}$, express the corresponding point on the hyperbola
  $$x:=F\left(\theta \right)\cosh\alpha +{2\;∥u∥}^{-1}\;u\sinh\alpha ;$$
3. Set the parameter by isometry $\xi :=F^{-1}\left(x\right)$.

Thus, the resulting parameter ξ is obtained as $\exp_{\theta }\left(u\right)$.

#### 4.2.3. Numerical Example

Now, we test the above algorithm for solving Karcher mean by a numerical example. The data set *S* is chosen as containing 10 empirical probability vectors from the first to the tenth 100 decimal digits of π.

To illustrate the goodness of each iteration, we present the norm of the negative gradient vector field at each iteration point via Equation (26), as shown in Table 4. Within each model, we present in column (a) the iteration results starting with the initial points chosen as the aforementioned way, while in column (b), the iteration results with initial points chosen as the usual Euclidean means are provided for comparison.

As we can see, all of the four iterations shown here converge rapidly within the first two steps, and our aforementioned choice for initial points is apparently better than the usual choice of Euclidean means. In conclusion, this example, to some extent, shows the effectiveness of our computation scheme for the Karcher mean within the multinomial and negative multinomial models.

## 5. Conclusions

In this paper, we have studied various information geometric properties based on divergence functions for the multinomial and negative multinomial models. Some pre-derived expressions of fundamental geometric quantities, such as Fisher-Riemannian metric, isometric representation and α-curvature, have made it clear that these two models can be put together into a complementary view. With the aid of these geometric structures, we investigate the average problems on these two models. We have proposed the conception of generalized Chernoff points as midpoints of two points and presented some determining equations for them. Then we provided an effective computation scheme for the Karcher mean of multiple points on the multinomial and negative multinomial models.

## Figures and Tables

**Table 1:** Complementary geometric structures.

Model	Isometric Space	Sectional Curvature	α-Sectional Curvature
$M_{N}$	*n*-sphere within nonnegative orthant	$\frac{1}{4\;N}$	$\frac{1-\alpha^{2}}{4\;N}$
${\mathrm{NM}}_{M}$	*n*-hyperbola within nonnegative orthant	$-\frac{1}{4\;M}$	$-\frac{1-\alpha^{2}}{4\;M}$

**Table 2:** Chernoff points of multinomial model (%).

	0	1	2	3	4	5	6	7	8	9
$\theta^{\prime }$	8.00	8.00	12.00	11.00	10.00	8.00	9.00	8.00	12.00	14.00
$\theta^{\prime \prime }$	11.00	12.00	12.00	8.00	12.00	12.00	7.00	4.00	13.00	9.00
$CP_{I}^{\left(1\right)}$	9.49	9.91	12.17	9.53	11.10	9.91	8.06	5.76	12.66	11.41
$CP_{I}^{\left(-1\right)}$	9.52	10.02	12.00	9.48	11.01	10.02	7.99	5.98	12.51	11.47
$CP_{\mathrm{II}}^{\left(1\right)}$	9.48	9.89	12.17	9.55	11.08	9.89	8.07	5.78	12.65	11.44
$CP_{\mathrm{II}}^{\left(-1\right)}$	9.54	10.05	12.00	9.46	11.02	10.05	7.98	5.95	12.51	11.44
$CP^{\left(0\right)}$	9.51	9.97	12.08	9.51	11.05	9.97	8.02	5.87	12.58	11.44

**Table 3:** Chernoff points of negative multinomial model (%).

	0	1	2	3	4	5	6	7	8	9
$\theta^{\prime }$	8.62	8.62	12.93	11.21	9.48	7.76	8.62	6.90	13.79	12.07
$\theta^{\prime \prime }$	10.99	12.09	10.99	6.59	14.29	12.09	6.59	4.40	12.09	9.89
$CP_{I}^{\left(1\right)}$	10.99	10.11	11.98	8.73	11.50	9.56	7.60	5.58	12.96	10.99
$CP_{I}^{\left(-1\right)}$	9.73	10.25	12.02	9.04	11.74	9.79	7.67	5.72	12.99	11.05
$CP_{\mathrm{II}}^{\left(1\right)}$	10.91	10.00	12.04	8.87	11.36	9.43	7.66	5.66	13.01	11.06
$CP_{\mathrm{II}}^{\left(-1\right)}$	9.80	10.35	11.96	8.90	11.88	9.92	7.61	5.65	12.94	10.98
$CP^{\left(0\right)}$	10.36	10.18	12.00	8.89	11.62	9.67	7.63	5.65	12.98	11.02

**Table 4:** Iteration for the Karcher mean illustrated by $∥-\nabla f∥.$

	$M_{N}$		${\mathrm{NM}}_{M}$	
Iteration	(a)	(b)	(a)	(b)
0	$8.21\times 10^{-5}$	$1.07\times 10^{-2}$	$3.05\times 10^{-3}$	$1.85\times 10^{-1}$
1	$4.62\times 10^{-7}$	$6.28\times 10^{-5}$	$1.74\times 10^{-4}$	$1.20\times 10^{-2}$
2	$2.68\times 10^{-9}$	$3.90\times 10^{-7}$	$1.01\times 10^{-5}$	$7.88\times 10^{-4}$
